# Effects of SuperUlam on Supporting Concentration and Mood: A Randomized, Double-Blind, Placebo-Controlled Crossover Study

**DOI:** 10.1155/2013/238454

**Published:** 2013-11-28

**Authors:** Jay K Udani

**Affiliations:** ^1^Medicus Research, LLC, 18250 Roscoe Boulevard, Suite 240, Northridge, CA 91325, USA; ^2^Northridge Hospital Integrative Medicine Program, Northridge, CA 91325, USA

## Abstract

*Background*. SuperUlam is a proprietary blend of natural ingredients aimed at supporting brain health. We aimed to evaluate the effect of SuperUlam on attention and mood in healthy adults. *Methods*. Twenty healthy individuals aged 35–65 were enrolled in this randomized, double-blind, placebo-controlled, crossover study. Study duration was 3 weeks and consisted of 3 visits. Measurement of cognitive function included computer-based testing of reaction time, complex attention, working memory, sustained attention, and executive functioning. Mood testing was performed via the profile of mood states (POMS) survey and the Chalder fatigue scale. * Results*. Cognitive function testing demonstrated a significant improvement from baseline in executive functioning, cognitive flexibility, reaction time, and working memory in the product group only (*P* < 0.05). When comparing the study product to placebo, the data demonstrated a significant decrease in tension, depression, and anger (*P* < 0.05). There was no significant difference between the product and placebo in the other measures of mood, including vigor, fatigue, confusion, and total mood disturbance. No adverse events were reported. *Conclusions.* Supplementation with SuperUlam is safe to consume with potential benefits to cognitive function and mood.

## 1. Introduction

In the past several years, much attention has been paid to products aimed at increasing energy, attention, and concentration. Products such as energy drinks, herbal supplements, and teas are often marketed to healthy individuals who wish to improve their cognitive health. Although popular and readily available, these products are not without risks. While consumption of one serving by a healthy adult may prove no harm, adverse cardiovascular and neurologic effects have been reported [1]. This has led to an increasing interest by consumers for safe products that can acutely increase concentration and memory.

*Ginkgo biloba *is a tree leaf extract commonly used as a memory and concentration enhancement. It has been investigated extensively both in patients with dementia or cerebral insufficiency as well as in healthy adults [2–5]. Plant extracts such as sireh (*Piper betle*) are used traditionally as stimulants throughout Asia. The spice turmeric (*Curcuma longa*), commonly found in Asian curries, has been described to have antioxidant and has neuroprotective effects [6]. A recent study demonstrated that consumption of curry-spiced foods was associated with increased Mini-Mental State Examination scores in healthy elderly individuals [6]. Pegaga (*Centella asiatica*, also known as gotu kola) and curry leaf (*Murraya koenigii*) have both been shown to have neuroprotective effects in laboratory research [7, 8]. In Ayurvedic medicine, pegaga is known as a rejuvenating herb for nerve and brain cells and is thought to increase intelligence, longevity, and memory [7, 8]. Both vitamin C and vitamin E have been reported to have potential benefits to cognitive function in elderly patients and those suffering from Alzheimer's disease [9, 10]. Ulam raja (*Cosmos caudatus*) is an annual plant found in tropical regions and is traditionally used in Malaysian culture for antiaging and vascular health. Extracts from this plant have been found to be high in proanthocyanidins, which are a class of flavanols that have antioxidant properties [11].

SuperUlam is a proprietary product containing extracts of sireh (*Piper betle*), turmeric (*Curcuma longa*), pegaga (*Centella asiatica*), curry leaf (*Murraya koenigii*), selasih (*Ocimum basilicum*), kesum (*Polygonum minus*), ulam raja (*Cosmos caudatus*), and vitamins C and E. We aim to evaluate the effects of this proprietary blend on areas of cognitive function and mood.

Cognitive testing is aimed at making quantitative assessments of cognitive capabilities and degree of high-level thinking. The CNS Vital Signs Systems is a computerized neurocognitive test battery comprised of seven tests: verbal memory, visual memory, finger tapping, symbol digit coding, the Stroop test, a test of shifting attention, and the continuous performance test. The results of the CNS Vital Signs System have been shown to be reliable and valid, compared with conventional neuropsychological tests upon which they are based [12].

There are a number of approaches toward testing of mood. POMS is a psychological rating scale used to assess transient, distinct mood states [13]. The moods included are tension, depression, anger, vigor, fatigue, confusion, and total mood disturbance. The Chalder fatigue scale is a self-rating measure for both physical and mental symptoms [14]. The scale consists of eight questions relating to physical symptoms and six questions relating to symptoms of mental fatigue. Items are scored in a Likert format, with response options ranging from “better than usual” to “much worse than usual.” Lower scores are preferable.

This randomized, double-blind, placebo-controlled crossover study aims to evaluate the effects of SuperUlam on cognitive function and mood in healthy adult subjects.

## 2. Methods

### 2.1. Subject Population

The study protocol and material were approved by an Institutional Review Board (Copernicus Group IRB, Cary, NC) and all subjects provided written informed consent prior to participation. This study was conducted in accordance with Good Clinical Practice Guidelines and the ethical principles of the Declaration of Helsinki. Healthy volunteers were recruited from the general population by online recruiting, advertising, and available clinical trial databases. Recruitment and interventions were conducted at the StayWell Research study site located in Northridge, California. The inclusion and exclusion criteria are outlined in Table 1.

### 2.2. Study Products

The intervention product tested was SuperUlam (Biotropics; Selangor, Malaysia). The product ingredients are listed in Table 2. The placebo, dispensed in a sensory identical, opaque capsule, was a combination of microcrystalline cellulose, calcium phosphate, and other inactive components. Both the study product and placebo product were provided by the sponsor and both were GMP certified.

### 2.3. Study Design

This was a randomized, double-blind, placebo-controlled, crossover study. Simple randomization was prepared using a computer program based on the atmospheric noise method, and sequential assignment was used to determine group allocation. Group allocation was placed in individually numbered envelopes to maintain blinding of all individuals and study staff. Study subjects attended three visits, consisting of a screening visit followed by two visits where product is administered. Subjects were screened at visit 1 to determine study eligibility. This included detailed history and physical examination, questionnaires on alcohol use and compliance, POMS testing, and CNSVS testing. Prior to discharge, subjects were provided standardized food to consume prior to the next visit and instructed to fast for 10 hours and sleep between 4.5 and 5.5 hours the night before the next visit. Subjects were also asked to refrain from consuming caffeine 24 hours prior to baseline visit. On visit 2, subjects were randomized to receive a single dose of either the SuperUlam product or placebo. Testing consisted of CNSVS testing, the Chalder fatigue scale, and POMS scoring. After baseline testing was completed, the subject consumed the study product in clinic, and testing was then repeated every hour for five hours. Standardized food was provided during this time. Between visit 2 and visit 3, subjects underwent a seven-day washout period of any related supplements or stimulants. Crossover of study product was performed at visit 3, where subjects who had received placebo at visit 2 would receive product at visit 3, and those who received product at visit 2 would receive placebo at visit 2. Otherwise, previsit preparation and visit procedures were conducted in a manner identical to visit 2. Product was only consumed in clinic and no product was dispensed for home consumption.

### 2.4. Outcome Measures

The primary objective of this study was to assess the effects of the SuperUlam product compared to placebo on the ability to improve attention and memory. Endpoints included cognitive function measurements, which were assessed using the computer-based CNS Vital Signs System (CNSVSS) (Morrisville, NC). The secondary objective of this study was to assess the effects of the SuperUlam product compared to placebo on mood state which were measured by the POMS and the Chalder scales.  Table 3 outlines the scoring system and definitions for the CNSVSS, POMS, and Chalder scales. The tertiary objective of this study was to evaluate the safety of SuperUlam. Safety endpoints included vital signs (temperature, blood pressure, pulse, and respiratory rate) and adverse event (AE) reporting.

### 2.5. Statistical Analysis

Paired sample *t*-tests were used for within subject means comparisons and independent sample *t*-tests for between group comparisons (placebo versus active group). Statistical analyses were performed using SPSS Base System ver. 1.8 (IBM, Chicago IL, USA). *P* values less than 0.05 were considered statistically significant.

## 3. Results

**Figure 1:**
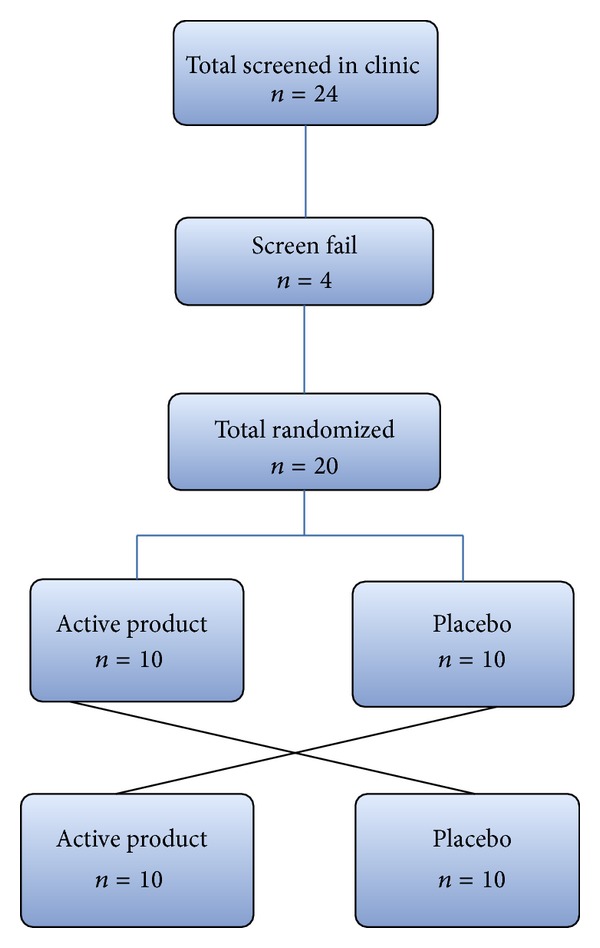
Study attrition chart.

Twenty-four subjects were screened to take part in this study. Of these, 20 were randomized and all 20 completed the study (Figure 1). Compliance was 100% for both placebo and product. Demographic data for the study participants is outlined in Table 4.

Baseline values for CNS Vital Signs testing found no significant difference between product and placebo groups. A significant increase in executive functioning was noted at hour 1 in the SuperUlam group compared with the placebo group (*P* = 0.047) (Table 5). Otherwise, there was no significant difference in any of the other components of CNS Vital signs testing between product and placebo.

When comparing hour 5 to baseline, subjects receiving active study product demonstrated statistically significant improvement in cognitive flexibility (*P* = 0.048), reaction time (*P* = 0.01), working memory (*P* < 0.01), sustained attention (*P* = 0.048), and executive functioning (*P* = 0.042). Subject in the active group did show an increase in processing speed from baseline to hour 5 (*P* < 0.01), a higher speed meaning that the subject took longer to take the test. The overall composite score or the average standard score across all tests showed a statistically significant improvement from baseline to hour 5 (*P* < 0.001).

When comparing hour 5 to baseline, subjects receiving the placebo product demonstrated statistically significant worsening in reaction time (*P* < 0.01) and processing speed (*P* = 0.047). Additionally, a significant worsening in working memory among the placebo group was noted when comparing baseline to hours 1 (*P* < 0.01), 2 (*P* < 0.01), 3 (*P* < 0.01), and 4 (*P* < 0.01). Subjects in the placebo group demonstrated improvement from baseline to hour 5 in sustained attention (*P* = 0.02). There was no statistically significant change in the average standard score between baseline and hour 5 in the placebo group.

Of the components of the POMS score at hour 5, when compared to placebo, the active product group demonstrated a significant improvement in depression (*P* < 0.01) and anger (*P* < 0.001). Otherwise, there was no significant difference in any of the other components of POMS scoring between product and placebo at hour 5.

When comparing hour 5 to baseline, subjects receiving active study product demonstrated statistically significant improvement in tension (*P* = 0.02), depression (*P* < 0.001), anger (*P* < 0.001), vigor (*P* < 0.001), confusion (*P* = 0.03), and total mood disturbance (*P* < 0.001). Subjects receiving placebo demonstrated significant improvement in anger (*P* < 0.001), vigor (*P* < 0.001), confusion (*P* < 0.001), and total mood disturbance (*P* < 0.001) (Table 6).

There were no significant differences between groups at any time point for the total physical or total mental scores on the Chalder fatigue scale (Table 7). There were no serious or other adverse events reported in this study, and safety laboratory parameters were not investigated.

## 4. Discussion

Interest in products aimed at improving cognitive performance and mood is steadily increasing. Younger individuals are interested in products that allow them to both study longer and increase energy for social life. As pharmaceutical agents are often plagued with side effects [15], many individuals are in search of a natural option for improving cognitive functioning and mood. Products containing *Ginkgo biloba* and natural sources of caffeine such as tea and guarana have been investigated in clinical trials and are readily commercially available [2–5, 16, 17]. Other products, such as energy drinks, may not be well researched, may be costly, may require large amounts, or may contain caffeine or other stimulants.

SuperUlam is a proprietary mixture of vitamins C and E as well as extracts from plants shown in laboratory research or traditional use to improve concentration, mood, and/or energy. Extracts of sireh (*Piper betle*), turmeric (*Curcuma longa*), pegaga (*Centella asiatica*), curry leaf (*Murraya koenigii*), selasih (*Ocimum basilicum*), kesum (*Polygonum minus*), and ulam raja (*Cosmos caudatus*) are included. Although the potential mechanisms of action for cognitive and mood improvements of these agents are not completely clear, there is evidence of neuroprotective effects of turmeric (*Curcuma longa*) and its constituent curcuminoids [18–21], as well as vitamins C and E (tocotrienols and tocopherols) [22–29] in animal models. Tocotrienols have been shown to reduce tumor necrosis factor-alpha and interleukin-1beta in brain regions of ethanol-treated animals [24]. A recent review described *in vitro *in cell culture studies that suggest the signal transduction mechanisms that may be involved in the neuroprotective effects of curcumin in turmeric (*Curcuma longa*) [30].

*Centella asiatica *is used traditionally in Ayurvedic and Chinese medicines as a brain tonic [7, 31–34]. The neuroprotective effects of *Centella asiatica *have been shown in various animal models [31, 32, 35], and the learning and memory-enhancing effects of a *Centella asiatica *extract were shown in a labyrinth test [36]. In an animal model, *Centella asiatica *also had antidepressant effects, reducing corticosterone levels and increasing levels of monoamine-related neurotransmitters [37].

*Murraya koenigii* (curry leaf extract) has also been shown to improve memory in animal models, perhaps by reducing cholinesterase activity [8, 38]. *Ocimum basilicum* has been traditionally used to support neuroprotection. An animal model has shown that extracts of *Ocimum basilicum* extract can improve memory and also have antioxidant effects [39]. Other constituents of SuperUlam with known antioxidant potential include *Polygonum minus* (kesum) [40], *Cosmos caudatus* (ulam raja), traditionally used to improve blood circulation, [11] and vitamins C and E [23, 25, 26, 41].

While this the first published human trial demonstrating the safety and efficacy of the proprietary SuperUlam product, evidence from other clinical trials demonstrates the effects of many of the individual ingredients found in SuperUlam. Early clinical evidence suggests improvement in cognitive function associated with *Centella asiatica* [42]. More recently, an aqueous extract of *Centella asiatica* standardized to constituent tannic acids, as well as asiaticoside and asiatic acid, improved cognitive performance in healthy men and women [43, 44] and in elderly subjects with mild cognitive impairment [45, 46].

In this study, SuperUlam was investigated for its ability to improve concentration and mood in healthy adults aged 35–65 years. Significant improvements were noted in multiple aspects of cognitive function and mood state. Of note, the data did demonstrate a worsening in processing speed in both active and placebo groups. We attribute this to fatigue related to performing the testing itself. This is of interest given that these subjects were all partially sleep deprived and had already experienced hours of testing. A loss of vigor and increased confusion would be expected at this stage. A reduction in confusion at this stage may be related to a higher level of mental clarity. Overall, the results suggest that SuperUlam improved concentration earlier in the study (approximately hours 1-2) and mood later in the study (hours 3–5) in healthy adults.

In conclusion, the SuperUlam product appears to have a neurostimulatory and neurocognitive performance enhancement effect, impacting various aspects of cognition and memory at different times between one and five hours after a single dose. The product also appears to induce a state of relaxation as evidenced by reductions in tension, anger, and depression between hour four and five after dosing. The results of this study suggest that this product may be of interest to generally healthy middle-aged adults.

We attribute the lack of statistical significance in our study to small sample size. A future higher-powered study is needed to investigate an appropriate number of individuals to generate further statistically significant results. Further animal and *in vitro *studies are needed in order to fully determine the mechanism of action. Pharmacokinetic and pharmacodynamic testing may be required to understand the dissolution, absorption, and activity characteristics of this product. There is also need for investigation in older individuals and young adults, as well as in adults using medications or with disorders that may increase the risk of cognitive impairment.

## Figures and Tables

**Table 1:** Inclusion and exclusion criteria.

Inclusion criteria	Exclusion criteria
Age between 35–65 years	Active or history of substance or alcohol abuse
BMI between 18–30 kg/m^2^	History of major depression, bipolar disorder, or schizophrenia
POMS score >15 at screening	Use of medications of ADD or ADHD
CNSVS score >6 at screening	Smoking
	Caffeine intake of over four servings daily (roughly 300–400 mg)
	Use of any drugs or dietary supplements that may affect memory or mental performance
	Pregnant or lactating
	Any medical condition which in the opinion of the investigator might interfere with the subject's participation in the trial

BMI: body mass index.

POMS: profile of mood states.

CNSVS: CNS vital signs.

**Table 2:** SuperUlam ingredients.

SuperUlam Capsule	
Extracts	
Sireh extract	150 mg
Turmeric extract	50 mg
Pegaga extract	100 mg
Curry leaf extract	50 mg
Selasih extract	50 mg
Kesum extract	150** **mg
Ulam Raja extract	25 mg
Tocobeads	25 mg
Ascorbic Acid	36 mg
Vitamin E 50% CWS/S	8 mg

Other Ingredients	
Microcrystalline cellulose PH102	194 mg
Calcium phosphate dibasic dihydrate	68 mg
Sodium starch glycolate	25 mg
Glyceryl behenate	37 mg
Colloidal silicon dioxide	27 mg

Coating	
Hydroxypropylmethylcellulose	14.9** **mg
Glycerin	5 mg
Chlorophyll, sodium copper complex	0.56 mg
Carnauba wax	0.12 mg
Titanium oxide	8.9 mg

**Table 3:** Scoring system for CNSVSS, POMS, and Chalder scales.

Scales and scoring	Definition	Units and normal range	Explanation
CNS vital signs			
Cognitive flexibility	Measure of frontal lobe functioning	Milliseconds Age-dependent	A higher value represents a higher level of functioning
Reaction time	Average measure of two distinct attention components	Milliseconds Age-dependent	A higher score represents a longer reaction time
Complex attention	Combined measure of simple, choice, and shifting attention elements	Milliseconds Age-dependent	A higher score represents a higher level of attention
Working memory	Measures well-known “2-back” working memory	Milliseconds Age-dependent	A higher score represents a higher level of working memory
Sustained attention	Combined measure of three cascading attention tests each slightly more difficult than the previous.	Milliseconds Age-dependent	A higher score represents a higher level of sustained attention
Executive functioning	Measure of how well a subject deals with making correct decisions in a shifting rule and target environment	Milliseconds Age-dependent	A higher score represents a higher level of executive functioning
Processing speed	Scores how well a subject deals with moving across a keyboard based on keys and stimuli	Milliseconds Age-dependent	A higher score represents a longer processing speed
Average standard score	Composite score of overall cognitive function	Milliseconds Age-dependent	A higher score represents a higher level of overall cognitive function

POMS scoring			
Tension	Assess transient, distinct mood state of tension	0–4 Likert scale No normal range	A higher score indicates that subject tends to be fidgety, restless, and quickly frustrated with people
Depression	Assess transient, distinct mood state of depression	0–4 Likert scale No normal range	A higher score indicates that subject feels complete and utter loss of hope and unable to carry out normal activities
Anger	Assess transient, distinct mood state of anger	0–4 Likert scale No normal range	A higher score indicates the subject's intensity of expressing anger at a particular time
Vigor	Assess transient, distinct mood state of vigor	0–4 Likert scale No normal range	A higher score indicates that the subject is feeling cheerful, lively, alert, active, and carefree
Fatigue	Assess transient, distinct mood state of fatigue	0–4 Likert scale No normal range	A higher score indicates more severe fatigue, fatigue distress, or impact on activities of daily living
Confusion	Assess transient, distinct mood state of confusion	0–4 Likert scale No normal range	A higher score indicates that subject tends to feel mixed up and confused either with instructions or surrounding environment
Total mood disturbance	Composite score of distressed mood states	0–4 Likert scale No normal range	A higher score indicates anxiety, emotional suppression, and psychological distress

Chalder scales			
Total physical	Self-rated 8-question scale of physical symptoms	0–3 Likert scale No normal range	High scores indicate an increased risk for cardiovascular diseases
Total mental	Self-rated 6-question scale regarding mental fatigue	0–3 Likert scale No normal range	High scores indicate presence of anxiety and depression

**Table 4:** Demographic data.

Baseline characteristics	Male	Female
*N*	10	10
Age (mean)	47.7	47.6
Age (range)	36–65	43–54
Weight in lbs (mean)	193.1	155.3
Weight in lbs (range)	169–216	120–190
BMI (mean)	27.2	26.4
BMI (range)	25–30	24–30
Menopausal %	NA	20%
Marital status		
Single	1	0
Married	7	8
Divorced	0	2
Separated	2	0
Widowed	0	0
Domestic partner	0	0
Ethnicity		
White	3	2
Asian	0	0
African-American	1	0
Hispanic/Latino	6	8
American Indian/Alaska Native	0	0
Hawaiian/Pacific Islander	0	0
Other	0	0

**Table 5:** Effect of treatment on CNS vital signs testing.

Units (ms)	Baseline (mean ± SE)		Hour 1 (mean ± SE)		Hour 2 (mean ± SE)		Hour 3 (mean ± SE)		Hour 4 (mean ± SE)		Hour 5 (mean ± SE)	
	SuperUlam	Placebo	SuperUlam	Placebo	SuperUlam	Placebo	SuperUlam	Placebo	SuperUlam	Placebo	SuperUlam	Placebo
Cognitive flexibility	115.25 ± 2.07	111.60 ± 3.37	116.45 ± 1.63	115.70 ± 3.75	112.35 ± 5.11	112.60 ± 3.60	118.90 ± 1.75	113.45 ± 3.42	119.75 ± 1.60	117.55 ± 2.26	117.25 ± 2.27	116.85 ± 2.37
Reaction time	104.25 ± 2.66	100.65 ± 3.24	101.95 ± 2.97	99.25 ± 3.24	100.45 ± 3.19	106.60 ± 3.76	103.25 ± 3.14	100.90 ± 2.98	100.90 ± 3.56	101.15 ± 3.65	101.60 ± 3.51	102.55 ± 2.90
Complex attention	106.65 ± 2.36	101.40 ± 3.94	108.75 ± 1.56	106.80 ± 3.22	101.50 ± 5.93	98.50 ± 5.39	106.70 ± 2.58	96.60 ± 5.68	110.15 ± 7.55	104.65 ± 3.15	106.20 ± 2.29	105.10 ± 3.50
Working memory	97.05 ± 2.79	97.95 ± 4.02	97.20 ± 2.58	97.50 ± 3.49	98.25 ± 3.11	95.75 ± 3.67	99.35 ± 2.99	96.70 ± 3.44	98.40 ± 3.80	96.55 ± 3.25	102.15 ± 3.06	95.35 ± 4.28
Sustained attention	97.25 ± 3.45	97.30 ± 4.08	97.75 ± 3.30	99.05 ± 3.27	97.65 ± 2.44	94.60 ± 4.16	96.15 ± 4.30	97.20 ± 3.35	99.90 ± 3.79	96.70 ± 3.72	97.25 ± 4.47	95.30 ± 3.98
Executive functioning	115.95 ± 2.03	112.45 ± 3.46	116.60 ± 1.60^a^	116.05 ± 3.80	112.85 ± 5.10	114.40 ± 3.42	118.95 ± 1.68	114.45 ± 3.43	120.15 ± 1.57	118.20 ± 2.19	117.90 ± 2.08	117.05 ± 2.26
Processing speed	108.70 ± 3.02	103.85 ± 2.98	108.35 ± 3.52	109.75 ± 3.81	115.75 ± 3.34	114.40 ± 3.08	113.60 ± 2.85	112.45 ± 3.72	117.25 ± 3.57	115.40 ± 2.96	117.90 ± 2.67	115.40 ± 3.62
Average standard score	106.46 ± 1.83	103.60 ± 2.48	106.72 ± 1.72	106.30 ± 2.46	105.54 ± 3.15	105.27 ± 2.34	108.15 ± 1.63	104.53 ± 2.67	109.51 ± 1.73	107.16 ± 2.08	108.62 ± 1.69	106.81 ± 1.82

^a^ *P* < 0.05 versus placebo at this time point.

**Table 6:** Effect of treatment on POMS.

POMS	Baseline (mean ± SE)		Hour 1 (mean ± SE)		Hour 2 (mean ± SE)		Hour 3 (mean ± SE)		Hour 4 (mean ± SE)		Hour 5 (mean ± SE)	
	SuperUlam	Placebo	SuperUlam	Placebo	SuperUlam	Placebo	SuperUlam	Placebo	SuperUlam	Placebo	SuperUlam	Placebo
Tension	0.30 ± 0.15	0.40 ± 0.18	0.25 ± 0.12	0.35 ± 0.15	0.10 ± 0.07^a^	0.35 ± 0.15	0.25 ± 0.16	0.70 ± 0.41	0.15 ± 0.11	0.35 ± 0.15	0.25 ± 0.12	0.20 ± 0.16
Depression	0.10 ± 0.10	0.20 ± 0.16	0.25 ± 0.25	0.20 ± 0.12	0.20 ± 0.20	0.10 ± 0.07	0.05 ± 0.05	0.15 ± 0.11	0.00 ± 0.00^b^	0.10 ± 0.10	0.00 ± 0.00^a^	0.10 ± 0.07
Anger	0.15 ± 0.11	0.05 ± 0.05	0.25 ± 0.18	0.35 ± 0.15	0.25 ± 0.18^b^	0.05 ± 0.05	0.00 ± 0.00^b^	0.15 ± 0.15	0.00 ± 0.00^b^	0.10 ± 0.10	0.00 ± 0.00	0.00 ± 0.00
Vigor	8.60 ± 1.30	8.15 ± 1.20	8.10 ± 1.26	7.00 ± 1.16	8.95 ± 1.42	8.00 ± 1.34	8.95 ± 1.39	7.70 ± 1.27	8.80 ± 1.41	8.55 ± 1.30	8.70 ± 1.42	8.00 ± 1.19
Fatigue	0.85 ± 0.35	1.00 ± 0.31	1.30 ± 0.65	1.00 ± 0.41	1.10 ± 0.67	0.90 ± 0.35	0.50 ± 0.19	0.80 ± 0.44	0.45 ± 0.20	0.75 ± 0.42	0.45 ± 0.20	0.45 ± 0.24
Confusion	2.55 ± 0.39	2.40 ± 0.30	2.30 ± 0.31	2.55 ± 0.34	2.50 ± 0.60	2.40 ± 0.30	2.10 ± 0.31	2.75 ± 0.44	2.25 ± 0.32	2.45 ± 0.36	2.35 ± 0.32	2.25 ± 0.30
Total mood disturbance	−4.65 ± 1.75	−4.10 ± 1.57	−3.75 ± 2.18	−2.55 ± 1.72	−4.80 ± 2.57	−4.20 ± 1.70	−6.05 ± 1.74	−3.15 ± 2.10	−5.95 ± 1.69	−4.80 ± 1.89	−5.65 ± 1.72	−5.00 ± 1.49

^a^ *P* < 0.01 versus placebo at this time point; ^b^ *P* < 0.05 versus placebo at this time point.

**Table 7:** Effect of treatment on the Chalder fatigue scale.

	Baseline (mean ± SE)		Hour 1 (mean ± SE)		Hour 2 (mean ± SE)		Hour 3 (mean ± SE)		Hour 4 (mean ± SE)		Hour 5 (mean ± SE)	
	SuperUlam	Placebo	SuperUlam	Placebo	SuperUlam	Placebo	SuperUlam	Placebo	SuperUlam	Placebo	SuperUlam	Placebo
Total physical	7.65 ± 0.70	7.50 ± 0.52	8.15 ± 0.96	8.10 ± 0.74	7.95 ± 1.10	7.15 ± 0.62	7.10 ± 0.87	7.35 ± 0.70	6.25 ± 0.86	5.85 ± 0.79	6.80 ± 0.78	6.35 ± 0.66
Total mental	5.15 ± 0.53	5.15 ± 0.47	5.25 ± 0.63	5.35 ± 0.52	5.35 ± 0.61	5.00 ± 0.52	4.90 ± 0.64	5.05 ± 0.48	4.20 ± 0.59	4.45 ± 0.59	4.65 ± 0.47	4.50 ± 0.56
